# Brain-inspired speech segmentation for automatic speech recognition using the speech envelope as a temporal reference

**DOI:** 10.1038/srep37647

**Published:** 2016-11-23

**Authors:** Byeongwook Lee, Kwang-Hyun Cho

**Affiliations:** 1 Laboratory for Systems Biology and Bio-inspired Engineering, Department of Bio and Brain Engineering, Korea Advanced Institute of Science and Technology (KAIST), Daejeon, 34141, Republic of Korea

## Abstract

Speech segmentation is a crucial step in automatic speech recognition because additional speech analyses are performed for each framed speech segment. Conventional segmentation techniques primarily segment speech using a fixed frame size for computational simplicity. However, this approach is insufficient for capturing the quasi-regular structure of speech, which causes substantial recognition failure in noisy environments. How does the brain handle quasi-regular structured speech and maintain high recognition performance under any circumstance? Recent neurophysiological studies have suggested that the phase of neuronal oscillations in the auditory cortex contributes to accurate speech recognition by guiding speech segmentation into smaller units at different timescales. A phase-locked relationship between neuronal oscillation and the speech envelope has recently been obtained, which suggests that the speech envelope provides a foundation for multi-timescale speech segmental information. In this study, we quantitatively investigated the role of the speech envelope as a potential temporal reference to segment speech using its instantaneous phase information. We evaluated the proposed approach by the achieved information gain and recognition performance in various noisy environments. The results indicate that the proposed segmentation scheme not only extracts more information from speech but also provides greater robustness in a recognition test.

Segmenting continuous speech into short frames is the first step in the feature extraction process of an automatic speech recognition (ASR) system. Because additional feature extraction steps are based on each framed speech segment, adequate segmentation is necessary to capture the unique temporal dynamics within speech. The most commonly used speech segmentation technique in state-of-the-art ASR systems is the fixed frame size and rate (FFSR) technique, which segments input speech with a fixed frame size by shifting it in a typical time order (conventionally a 25 ms frame with a 10 ms shift) (Fig. 1, top)^1^. Although the FFSR provides excellent speech recognition performance with clean speech, recognition performance rapidly degrades when noise corrupts speech. Degradation of the recognition performance is primarily attributed to the notion that the FFSR is incapable of adapting to the quasi-regular structure of speech. The conventional frame size of 25 ms becomes insufficient because it can smear the dynamic properties of rapidly changing spectral characteristics within a speech signal, such as the peak of the stop consonant^2^ or the transition region between phonemes^3^,^4^. Furthermore, the conventional frame shift rate of 10 ms is too sparse to capture the short duration attributes of a sufficient number of frames. As a result, the peaks of the stop consonant or transition period are easily smeared by noise, which causes recognition failure. Conversely, for the periodic parts of speech, such as a vowel, the conventional frame size and shift rate cause unnecessary overlap, leading to the addition of redundant information and insertion errors in noisy environments^5^. To overcome these problems, various speech segmentation techniques have been proposed^6^. The variable frame rate (VFR) technique is the most widely employed scheme as a substitute for the FFSR scheme^4^,^5^,^7^. The VFR technique is done by extracting speech feature vectors with the FFSR scheme and determining which frame to retain. Such technique has been shown to improve performance in clean and noisy environments compared with the FFSR scheme. Yet, it needs to examine speech at much shorter intervals (e.g., 2.5 ms), which requires repetitive calculations of the predefined distance measures and frame selections between adjacent frames, producing high computational complexity^4^,^7^.

Although an ASR system struggles with noisy environments, the human auditory system maintains high speech recognition performance in various circumstances. The cause of the noise robustness in the system remains ambiguous; however, the way that the auditory system segments continuous speech is a critical factor in noise robustness speech recognition^8^,^9^. Although speech segmentation is easily performed in an ASR system with the use of external time bins as guides, the brain, which does not have an external temporal reference, has to segment continuous speech by relying on an intrinsic timing mechanism. The intrinsic reference for speech segmentation in the brain and its robustness against noise have remained a central question in neuroscience. Recent studies have suggested that different frequency band neuronal oscillations, which fluctuate in the auditory cortex, create a *nested oscillatory reference* that integrates information across different timescales in a hierarchical and interdependent manner to participate in segmenting continuous speech^8^,^9^,^10^,^11^,^12^,^13^. Neurophysiological analyses have demonstrated that *such nested oscillatory reference* could provide more than one timescale to capture information from speech (a timescale that is appropriate for processing short duration parts, such as a consonant or transition versus a timescale that is appropriate for processing relatively longer duration parts, such as a vowel)^13^. During speech comprehension, low-frequency ranges of the speech envelope are phase-locked to the low-frequency ranges of neuronal oscillations in the auditory cortex^14^,^15^,^16^,^17^,^18^,^19^,^20^. This close correspondence between the phase of the speech envelope and the neuronal oscillations suggests the hypothesis that the phase of the speech envelope inherits information that provides a temporal reference to segment speech. Previous studies have supported this hypothesis by demonstrating the dependence of speech intelligibility on the speech envelope; manipulating the temporal modulation of speech to unnaturally fast or slow rates, which eventually corrupts the temporal dynamics of the speech envelope, caused serious degradation in intelligibility, although the fine structure was preserved^21^,^22^. In an extreme case, when temporal modulation of speech was completely eliminated, intelligibility was reduced to 5%^23^,^24^. This finding can be explained by a lack of reference, which is supposed to segment speech and place the spectral structure contents into their appropriate context. As a potential mechanism of the multi-timescale speech segmentation, phase partitioned (speech-induced) neuronal oscillation was proposed to serve as a potential reference to partition speech into smaller units over the scale of tens to hundreds of milliseconds (Fig. 1, bottom)^8^,^9^,^25^,^26^,^27^.

In this study, we tested the hypothesis that the speech envelope serves as a potential temporal reference for segmenting a continuous speech signal into smaller units by considering the temporal dynamics of various levels of linguistic units. We created a *nested oscillatory reference* by extracting and nesting two sub-band oscillations from the speech envelope, namely, the primary and secondary frequency band oscillations. The instantaneous phase values of those two sub-band oscillations are extracted, and the time points that the phase value crosses the predetermined phase boundaries are used to represent the start and end points for the speech segmental reference. Speech is segmented using primary frequency band oscillation and re-segmented with secondary frequency band oscillation if the speech segments obtained by the primary frequency band oscillation satisfy the predetermined criteria. In this study, six typical frequency bands under 50 Hz (i.e., delta, 0.4~4 Hz; theta, 4~10 Hz; alpha, 11~16 Hz; beta, 16~25 Hz; low gamma, 25~35 Hz; and mid gamma, 35~50 Hz) of the speech envelope were examined as potential frequency bands of primary and secondary band oscillations. These frequency bands were chosen because they not only have a close correspondence with the timescales of various units in speech^28^,^29^,^30^ (e.g., sub-phonemic, phonemic, and syllabic) but are also extensively observed in the brain cognitive processes, including speech comprehension at the auditory cortex^22^,^23^,^24^. The various combinations of primary and secondary frequency band oscillations were compared to obtain the optimal *nested temporal reference*, which serves as the highest information extraction reference for speech. We named the proposed speech segmentation technique the Nested Variable Frame Size (NVFS) technique because the frame size is flexibly determined by the instantaneous phase of two nested oscillatory references.

In the experiments, syllable unit signals, which are composed of stop consonants and vowels, were employed. Stop consonants and vowels exhibit distinct disparity in their temporal dynamics; the stop consonant is the shortest and most aperiodic phoneme type, whereas the vowel is the longest and most periodic phoneme type. We expected that these distinct differences would maximize the result of our approach by applying different sizes and numbers of frames to capture the spectral changes of each phoneme class. The stop consonant accounts for more than 35% of the error relative to other phoneme classes during recognition in a noisy environment^31^. Therefore, increasing the noise robustness of stop consonant recognition is necessary to increase the total recognition robustness of an ASR system. We quantitatively compared the amount of information extracted by the proposed NVFS scheme with the conventional FFSR scheme and compared the effectiveness of each segmentation scheme with a speech recognition test.

## Results

**Figure 2:**
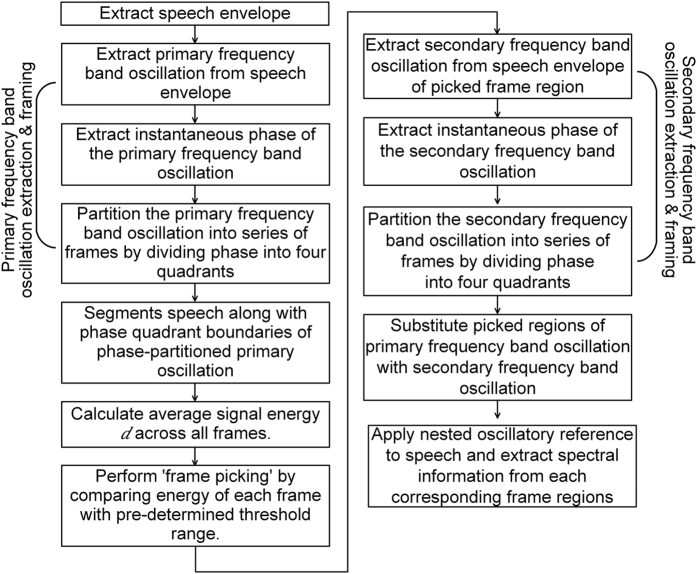
Flow chart of proposed speech segmentation scheme. A flow chart that shows the computation of the speech segmentation boundaries for the NVFS scheme.

During speech comprehension in the brain, the presence of important events is indicated by the changes in the instantaneous phase of nested neuronal oscillations^9^,^32^. By following these observations in the brain, the nested oscillatory reference effect in the auditory system is modeled by a series of steps as follows: (i) extract primary and secondary frequency band oscillations from the speech envelope as speech segmental references; (ii) partition primary and secondary frequency band oscillations using their phase quadrant boundaries as the frame start and end points, and (iii) couple primary and secondary frequency band oscillations such that the property of the primary frequency band oscillation shapes the appearance of the secondary frequency band oscillation. If the energy of the framed speech segment created by the primary frequency band oscillation falls within the pre-determined threshold range, it substitutes the oscillatory reference of the corresponding region with the secondary frequency band oscillation (refer to Methods for details on creating a nested oscillatory reference). A flow chart that describes the computation of the NVFS scheme is shown in Fig. 2.

### An example of the NVFS segmentation scheme

**Figure 3:**
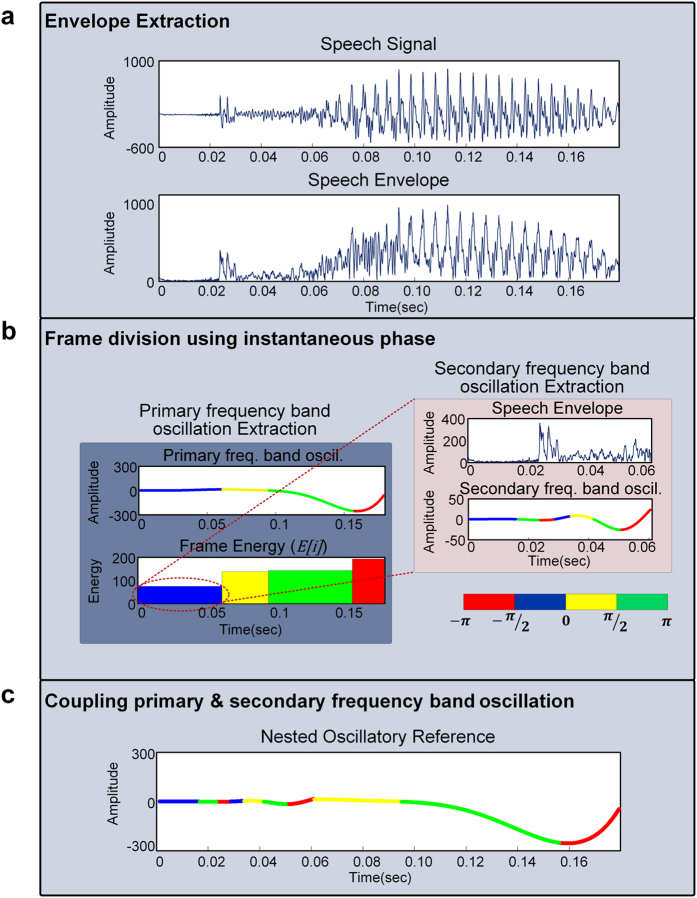
Nested oscillatory reference extraction process. (**a**) The waveform and envelope of a /pa/ utterance. (**b**) Frame division according to the phase quadrants of the extracted primary and secondary frequency band oscillations. (**c**) Resulting nested oscillatory reference.

Figure 3 shows how the frame boundaries are chosen by the proposed NVFS scheme for the signal/pa/ spoken by a male speaker. Figure 3(a) shows the speech waveform and its envelope. The primary frequency band oscillation (in this study, 4~10 Hz) is extracted from the speech envelope. The oscillation is plotted with four different colors. The color of the line at each time denotes the four phase quadrants of the instantaneous oscillation phase (left part of Fig. 3(b)). Extraction of the secondary frequency band oscillation (in this study, 25~35 Hz) from the speech envelope is performed in the first frame region, where its energy falls within the threshold range (refer to Methods for details). The extracted secondary frequency band oscillation is also colored according to its instantaneous oscillation phase, as shown in the right part of Fig. 3(b). The first frame region of the primary frequency band oscillation is substituted with the secondary frequency band oscillation to create a nested oscillatory reference. The nested oscillatory reference, which serves as a temporal reference for segmenting the speech signal, is shown in Fig. 3(c).

### Measuring Mel-scaled entropy

**Figure 4:**
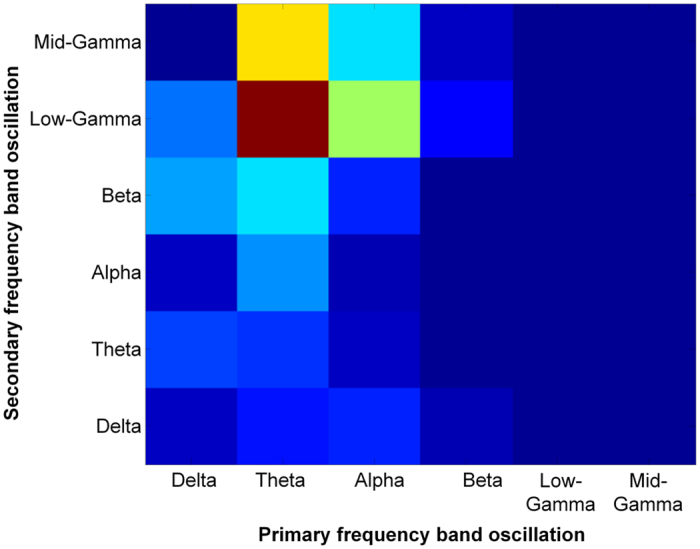
Optimal combination of primary and secondary frequency band oscillation. Preference distribution analysis for determining the optimal combination of the primary and secondary frequency bands that extract as much information as possible from each speech sample. Theta (4~10 Hz) as the primary frequency band oscillation and low gamma (25~35 Hz) as the secondary frequency band oscillation were chosen as the optimal combination for the temporal reference.

The speech envelope is composed of multiple frequency bands, which indicates that the envelope contains various timescales that deliver different speech features. Among the various temporal modulation rates of the speech envelope, only slow modulation rates (<50 Hz) are likely to be preferred by the auditory system in the brain^22^,^23^,^24^. In this study, the six typical frequency bands (i.e., delta, 0.4~4 Hz; theta, 4~10 Hz; alpha, 11~16 Hz; beta, 16~25 Hz; low gamma, 25~35 Hz; and mid gamma, 35~50 Hz) of the speech envelope are examined to serve as the primary and secondary frequency band oscillations to organize the *nested oscillatory reference*. The effectiveness of the created nested oscillatory reference as a temporal guide to segment speech was measured by calculating the cochlea-scaled spectral entropy (CSE)^33^, which represents the potential information gain from segmentation (refer to Methods for details). Greater unpredictability in speech is reflected by an increase in the CSE, which can be interpreted as providing potential information. We searched for dominant frequency band combinations of primary and secondary frequency band oscillations that form as a nested oscillatory reference to provide the highest CSE. We subsequently compared the CSE value of the proposed NVFS scheme with other segmentation schemes to determine which segmentation scheme can extract more information from speech. A total of 1542 samples from a test set in Table 1 are employed to identify the dominant primary and secondary frequency band oscillation combinations. We plotted the analysis results of the preference distribution of the primary and secondary frequency band combinations that maximize the CSE (Fig. 4). For the majority of the samples, the theta (4~10 Hz) range and low gamma (25~35 Hz) range of the speech envelope participated as the primary frequency band oscillations and secondary frequency band oscillations, respectively, in speech segmentation to yield the highest CSE. We assumed the theta and low gamma band oscillations as the optimal combination of the primary and secondary frequency band oscillations and further investigated the effectiveness of the NVFS scheme.

**Figure 5:**
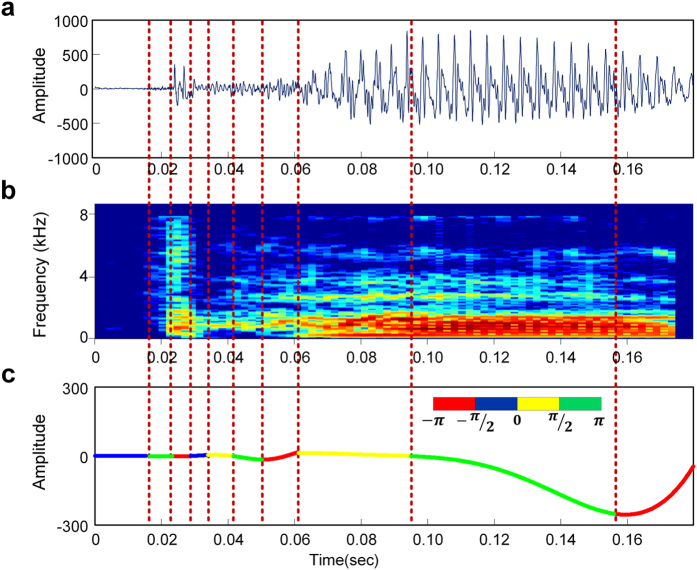
Example of the NVFS segmentation scheme. Example of (**a**) Waveform of a/pa/utterance in Fig. 3. (**b**) Corresponding spectrogram of the signal. (**c**) Generated nested oscillatory reference. The waveform and its corresponding spectrogram are divided by the phase quadrant boundaries of the nested oscillation (**c**).

To qualitatively assess the effectiveness of the theta and low gamma combination, we segmented the speech signal in Fig. 3 with the theta-low gamma nested oscillatory reference. The results are presented in Fig. 5. Figure 5(a) and (b) show the waveform of the speech and the spectrogram of the speech, respectively. The speech waveform and its corresponding spectrogram are divided by the phase quadrant boundaries of the nested oscillatory reference in Fig. 5(c). Note that the consonant and transition regions are captured with short length frames, whereas the vowel regions are captured with relatively long length frames. For this example, a total of six of nine frames are assigned to the consonant and transition regions, whereas three frames capture the vowel.

### Comparison of the NVFS with various segmentation schemes

**Figure 6:**
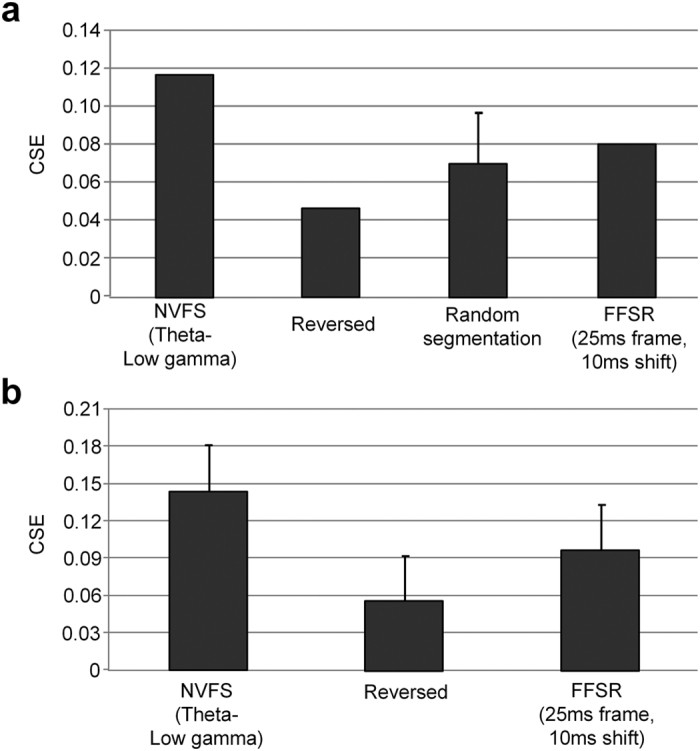
Calculation of information gain by various speech segmentation scheme. (**a**) The CSEs of various speech segmentation schemes are compared using the speech signal in Fig. 3. The CSE of the NVFS scheme is compared with the CSE of the (i) reversed order, (ii) random segmentation over 1000 trial, and (iii) conventional FFSR scheme (25 ms frame and 10 ms shift). (**b**) CSE comparison of 1542 samples from the test set.

To verify the significance of the NVFS scheme, we compared the potential information gain that was obtained by different segmentation schemes. First, we quantified the effectiveness of the NVFS scheme against other segmentation schemes by calculating the CSE of speech in Fig. 5. The CSE of the NVFS scheme was compared with the CSE of (i) the reversed frame order of the NVFS, in which the segmentation reference obtained by the NVFS is reversed back and forth, (ii) random segmentation in which the average CSE over 1000 repetitions of random segmentations, into nine frames (the same number of frames extracted by the NVFS for speech in Fig. 5), and (iii) the FFSR scheme (i.e., 25 ms frame with a 10 ms overlap), which is the conventional paradigm of the speech segmentation scheme in ASR. Figure 6(a) shows the results of the analysis. The theta-low gamma nested oscillation has the highest CSE, which suggests that this type of segmentation scheme can effectively extract information from the speech signal. Additional analysis was performed with the 1542 samples from the test set as in previous experiments. Each speech signal was segmented based on the NVFS scheme (theta-low gamma combination), reversed frame order of the NVFS, and the FFSR scheme (25 ms frame and 10 ms shift). The CSE for each segmentation scheme was averaged over all samples (Fig. 6(b)). The results indicate that the NVFS (theta-low gamma combination) scheme provides the highest information gain.

### Evaluation of speech recognition performance in noisy environments

**Figure 7:**
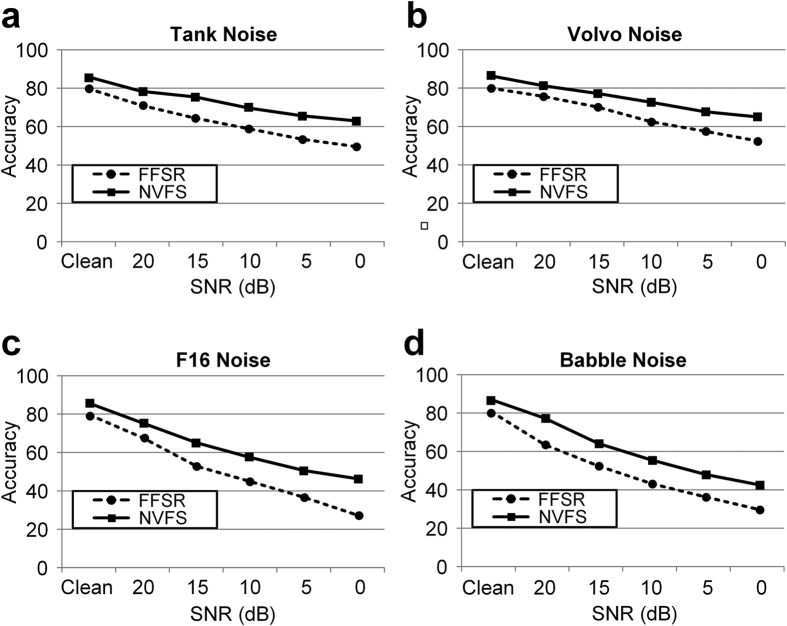
Comparison of speech recognition test between NVFS scheme and FFSR scheme. Recognition results obtained with four different additive noise conditions are plotted for various SNR levels. Graphs indicate that NVFS-based speech segmentation scheme provides more robust speech recognition then FFSR-based speech segmentation scheme.

**Figure 8:**
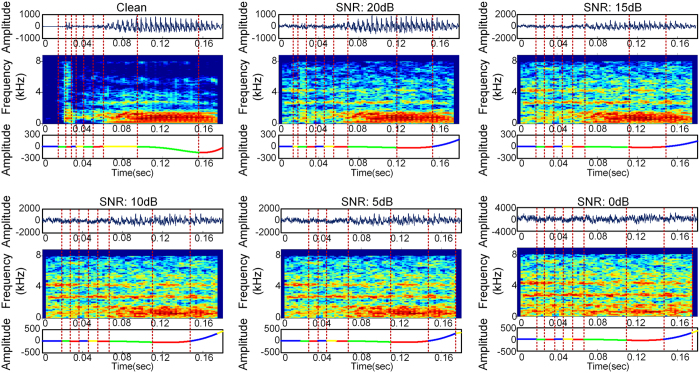
Noise robustness of nested oscillatory reference in various noise levels. Nested oscillatory reference extracted from the speech envelope is stably maintained over various noise levels.

We examined whether the superiority of the NVFS scheme in terms of information gain over the FFSR would manifest in the recognition performance. The average recognition performance of the NVFS based segmentation scheme was compared with the performance of the FFSR based segmentation scheme (i.e., 25 ms frame and 10 ms shift). The two speech segmentation schemes used the same feature extraction method and classifier—the Mel-Frequency Cepstral Coefficients and Hidden Markov Model, respectively (refer to Methods for details). Because the feature extraction and classification process after speech segmentation are equivalent, the performance gap between the NVFS scheme and FFSR scheme is solely dependent on how the speech is segmented. We examined the recognition performance of NVFS and FFSR in various noise types. The recognition results revealed a distinct performance gap between the two segmentation schemes (Fig. 7). In the distorted speech with various additive noise, the NVFS scheme showed a significantly better performance over the FFSR scheme with an average improvement of approximately 12%. The recognition performance for the different additive noise types is shown in Table 2 (see Supplementary Notes for the relationships between NVFS’s frame division criteria and its recognition performance). We determined that the generated nested oscillation by the NVFS scheme maintained high robustness as a reference signal in noisy environments. Figure 8 shows the alterations in the phase quadrant boundaries of the nested oscillatory reference with F16 cockpit noise for various SNRs. The frame boundaries remained nearly unchanged for all SNR levels, which shows the robustness of the phase-based frame boundaries (refer to Fig. S3 for more examples). The improvement in the recognition performance is attributed to the notion that the frame boundaries remain nearly unchanged over all SNR levels.

## Discussion

In this paper, we proposed a novel frame segmentation algorithm that was inspired by the speech processing mechanism of the brain. In the proposed method, nested sub-band oscillations extracted from the speech envelope are distinguished into separate frames according to their phase quadrant boundaries. A series of unequal size frames provides the efficient capture of each phonetic characteristic over time. In particular, short (e.g., 5~10 ms) frames are densely positioned around the consonant and transition regions, whereas only a few long (e.g., 20~50 ms) frames are placed around the steady part of the signal, such as vowels. We evaluated the benefits of the NVFS scheme over the conventional FFSR scheme. First, the CSE measure exhibited higher information gain from the NVFS than from the FFSR scheme. Second, a comparison of the speech recognition performance between the NVFS scheme and FFSR scheme indicated that the NVFS scheme had significant advantages over the FFSR scheme in various noise environments and SNR levels. These results suggest that the NVFS scheme provides not only higher information gain but also greater robustness compared with the FFSR scheme. The NVFS scheme is computationally efficient because the frames are naturally determined according to the phase quadrant boundaries of the nested oscillatory reference. As a result, the brain-inspired NVFS scheme provides an effective and computationally efficient speech segmentation scheme.

Our analysis revealed that the use of the theta-low gamma nested oscillation that was extracted from the speech envelope is sufficient as a temporal reference for speech segmentation from syllable units to sub-phonemic units of consonant and vowel. Previous studies have investigated the relationship between the theta range (4~10 Hz) speech envelope and the rates of various linguistic units^15^,^16^,^25^,^34^,^35^. The power of the theta range speech envelope arose from the opening of the jaw for voicing, which is an event that occurs at the beginning of every syllable during speech production. These results suggested that syllabic information is dominated in the theta range of the speech envelope. The emergence of the theta-low gamma nested timescale agrees with previous experimental findings. Giraud *et al.*^8^ insisted that cortical activity does not track whole speech modulation frequencies equally, but selectively tracks the theta and gamma range of speech modulation. The reason for this preferential tracking in the theta and gamma range can be explained by its potential role as a temporal reference to decompose from the syllable unit to the consonant and vowel unit. Perez *et al.*^13^ examined optimal timescales for the brain to discriminate consonant and vowel sounds by comparing neural discrimination with behavior discrimination. The results indicated that consonant and vowel discrimination are highly correlated with neural discrimination when different timescales of 1–20 ms and 40–100 ms are used to encode spike activities. These timescales are nearly consistent with the frame size that is provided by our results, which suggests that a nested theta-low gamma oscillatory reference is employed to provide different timescales to encode consonant and vowels. These findings suggest that the theta and gamma range of the speech envelope is a crucial ingredient that serves as a reference for decomposing speech from a syllable unit into phoneme units and extracting sub-phonemic features.

The phase of the neuronal oscillation seems to be involved in many cognitive processes due to its robustness against various noises. In the auditory cortex, the phase patterns of neuronal oscillations provide complementary information that cannot be provided by spectral information in spike trains^8^,^9^,^32^,^36^,^37^,^38^. Wang *et al.*^38^ demonstrated that the phases of typical frequency-range neuronal oscillations themselves provide distinct information to distinguish each phoneme, for which researchers succeeded in recognizing eight English phonemes with 66.7% accuracy using only the phase pattern. In circumstances in which the phases of the speech envelope and neuronal oscillations are locked, use of the phase information of the speech envelope is appealing as a new feature that can add complementary information with the spectral analysis. In the ASR field, however, only a few attempts have been made using the speech envelope as a new feature for ASR^39^,^40^,^41^,^42^. No attempts have been made to use the speech envelope as a temporal reference to directly segment speech. Our results suggest that a particular sub-band speech envelope provides a sufficient temporal reference to segment the speech signal.

## Methods

### Algorithm

In this section, we present the algorithm of the NVFS scheme to obtain a series of unequal size frames that specifically capture a set of sub-phonemic units. This algorithm is hierarchically employed by segmenting the syllable with the primary timescale and segmenting the specific region of the syllable (in this paper, consonant and transition) with the secondary timescale. The speech envelope is extracted using a Hilbert transform^43^. Theoretically, the analytic signal *s(n*) can be expressed as


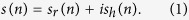


In this paper, *s*_*r*_(*n*) is the original speech signal, *s*_*h*_(*n*) is the Hilbert transform of *s*_*r*_(*n*), and 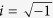. Eq. (1) can be rewritten in a polar coordinate system as


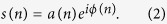


where the Hilbert envelope *a(n*) is the magnitude of the analytic signal:


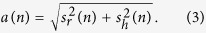


Primary frequency band oscillation, which serves as the primary timescale for the segmentation reference, is extracted from the envelope using third-order Butterworth filters. The instantaneous phase of the primary frequency band oscillation is subsequently determined from Eq. (2) as described in Eq. (4):


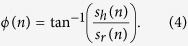


The phase of the primary frequency band oscillation is divided into four phase quadrants ([−π, −1/2*π], [−1/2*π, 0], [0, 1/2*π], [1/2*π, π]). Then, the quadrant boundaries are identified within the oscillation, which are subsequently employed as the frame start and end points for the speech segmentation. After partitioning the primary frequency band oscillation using its instantaneous phase, *s*_*r*_(*n*) is segmented into a series of frames by the phase quadrant boundaries of the primary frequency band oscillation. We calculate the energy of the i^th^ frame as


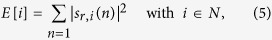


where *N* is the total number of frames. The average signal energy *d* is calculated across all frames and simple frame picking, which is based on the thresholding technique described in Eq. (6), is employed to detect the consonant region within the syllable


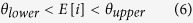


where 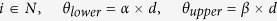. The thresholding with the lower threshold *θ*_*lower*_ and the upper threshold *θ*_*lower*_ distinguishes the consonant and transition regions from the salient and vowel regions, respectively. To estimate the optimal values of the two constant parameters *α* and *β* for the thresholding, we employed the genetic algorithm^44^ to minimize the sum of the squared difference between the consonant boundaries provided by the TIMIT database and the consonant boundaries obtained by applying a particular *α* and *β* to our algorithm. We chose 0.32 and 0.8 as optimal values for *α* and *β*. The frame regions of the primary frequency band oscillation within the threshold boundaries are substituted into the secondary frequency band oscillation, which is extracted from the speech envelope of the corresponding frame region. The procedures for the secondary frequency band oscillation are the same as the procedures for the extraction and frame partition of the primary frequency band oscillation: the instantaneous phase is extracted from the extracted sub-band oscillation, and the oscillation is divided according to the phase quadrants. Following this procedure, the nested oscillatory reference for speech segmentation is created.

### Data set construction

All experiments were conducted with the Texas Instruments/Massachusetts Institute of Technology (TIMIT) database^45^. The database contains 6,300 sentences recorded by 630 speakers from eight dialects. Because the ‘sa’ dialect sentences cause bias in the recognition results, we excluded these sentences from the experiment and used the remaining 5,040 sentences^46^. Among the 5,040 sentences, 3,696 utterances are obtained from the training set, and 1,344 utterances are obtained from the test set. The corpus that was used in the experiment was the syllable unit (CV), which is composed of six stop consonants (/p/, /t/, /k/, /b/, /d/, /g/) and three types of vowels: /aa/, (as in ‘bob’); /ih/, (as in ‘bit’); and /ah/, (as in ‘but’). The CV unit corpuses extracted from 5,040 sentences for each stop consonant type are listed in Table 1. A total of 6,037 CV unit speech data were extracted: 4,495 samples from the training set and 1,542 samples from the test set, respectively.

### Cochlea scaled spectral entropy

The cochlea-scaled spectral entropy (CSE) measures the relative unpredictability of speech by calculating the spectral differences between each frame. Framed speech segments were passed through 33 equivalent rectangular bandwidth (ERB) filter banks, and the spectral differences between adjacent frames were computed by calculating the Euclidean distance across normalized 33-filter bank channel outputs. The calculated Euclidean distances over all successive frames were averaged, and the averaged distance was taken as the measure of the CSE.

### Speech recognition test

The feature vectors were obtained by standard 13 Mel-Frequency Cepstral Coefficients (MFCCs) with their first- and second-order temporal derivatives, which produced a total 39 feature dimensions. The recognition performance was measured by the HMM classifier using the HTK toolkit^47^, in which each HHM model had four states and five Gaussian mixtures per state. The training set and test set in Table 1 were employed for the training and testing, respectively, of the acoustic model in various noise environments. Four different types of noise from the NOISEX-92 database^48^ were added to the clean test data at signal-to-noise ratio (SNR) levels of 20 dB to 0 dB in 5 dB decrements using the standard FaNT tool^49^. The noise types that were employed in the experiment were as follows: speech babble noise, F16 cockpit noise, Volvo car interior noise, and tank noise.

## Additional Information

**How to cite this article**: Lee, B. and Cho, K.-H. Brain-inspired speech segmentation for automatic speech recognition using the speech envelope as a temporal reference. *Sci. Rep.* **6**, 37647; doi: 10.1038/srep37647 (2016).

**Publisher’s note:** Springer Nature remains neutral with regard to jurisdictional claims in published maps and institutional affiliations.

## Supplementary Material

Supplementary Information

## Figures and Tables

**Figure 1:**
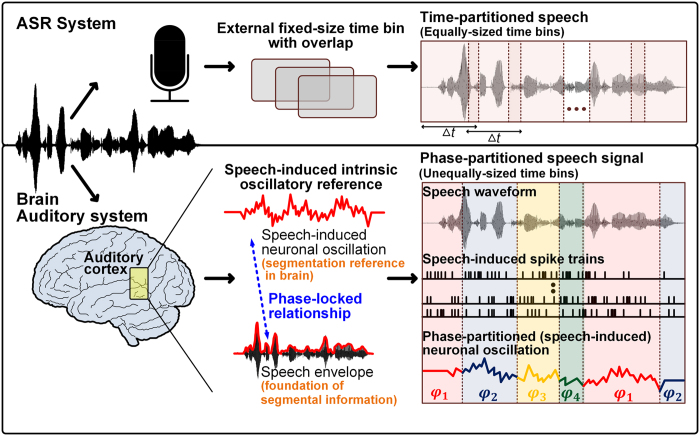
Schematic of speech segmentations in automatic speech recognition (ASR) system and brain. Segmenting continuous speech into short frames is the first step in the speech recognition process. In the ASR system, the most widely used speech segmentation approach employs fixed-size external time bins as a reference (‘time-partitioned’). This approach is computationally simple but has a limitation with respect to reflecting a quasi-regular structure of speech. Alternatively, the brain, which does not have an external timing reference, uses an intrinsic slow (neuronal) oscillatory signal as a segmentation reference. This oscillatory signal is phase-locked with the speech envelope during comprehension, which enables the reflection of quasi-regular temporal dynamics of speech in segmentation. The phase of this oscillatory signal is separated into four phase quadrants (φ_i_). The speech waveform and speech-induced spike trains are segmented and color-coded by the phase angle of the reference oscillatory signal (‘phased-partitioned’). This segmentation approach can potentially generate unequally sized time bins depending on the temporal dynamics of speech. In this paper, we investigated whether the speech envelope can serve as a potential temporal reference for segmenting speech.

**Table 1:** TIMIT composition of CV set used on training and test period.

Set	Voiceless Stop			Voiced Stop			Total
	/p/	/t/	/k/	/b/	/d/	/g/	
Training	526	1097	1059	630	901	282	4495
Test	215	341	350	228	291	117	1542

**Table 2:** Recognition results of each SNR level under four different additive noise conditions.

Noise Type	SNR (in dB)	FFSR	NVFS
Tank	20	71.3	78.4
	15	64.4	75.5
	10	58.8	69.8
	5	53.4	65.6
	0	49.7	62.9
	Average	59.5	70.4
Volvo	20	75.8	81.1
	15	70.5	77.2
	10	63.3	72.9
	5	58.7	68.3
	0	53.6	65.5
	Average	64.4	73.2
F16	20	67.6	75.6
	15	52.4	65.2
	10	44.2	57.3
	5	35.7	50.2
	0	26.1	45.8
	Average	45.3	58.8
Babble	20	63.4	76.7
	15	52.6	63.8
	10	43.4	55.3
	5	36.7	48.2
	0	30.2	42.7
	Average	43.6	57.3
