# Supplementary material for: Brain-inspired speech segmentation for automatic speech recognition using the speech envelope as a temporal reference
Source: Sci Rep. 2016 Nov 23;6:37647. doi: 10.1038/srep37647 (PMC5120313; doi:10.1038/srep37647)
Supplement: Supplementary Information [file srep37647-s1.pdf]

Brain-inspired speech segmentation for automatic speech recognition using the speech envelope as a temporal reference

Byeongwook Lee and Kwang-Hyun Cho*

Laboratory for Systems Biology and Bio-inspired Engineering, Department of Bio and Brain Engineering, Korea Advanced Institute of Science and Technology (KAIST), Daejeon, 34141, Republic of Korea.

Supplementary Information

*Corresponding author. E-mail: ckh@kaist.ac.kr, Phone: +82-42-350-4325, Fax: +82-42-350-4310, Web: http://sbie.kaist.ac.kr/

I. Supplementary Notes

Dependence of the algorithm on the number of division

We chose quadrant division for theta and gamma oscillations because the frame size obtained by this division is generally consistent with the optimal size that represents the spectral characteristics spread over the speech signals. The frame size obtained by dividing theta band (4~10 Hz) oscillations or low-gamma band (25~35 Hz) oscillations into quadrants ranges from 25 ms to 60 ms or 5 ms to 10 ms, respectively. These ranges are consistent with the ranges from previous speech recognition studies that were considered to be optimal for achieving a high recognition performance[1]. The use of two divisions produces a frame size that ranges from 50 ms to 120 ms (for theta band oscillation) or 10 ms to 20 ms (for low-gamma band oscillation), which is relatively large and can smear the temporal change of spectrum within the phoneme and between two phoneme boundaries. The use of eight divisions or denser divisions produces a relatively short frame size that ranges from 12.5 ms to 30 ms (for theta band oscillation) and 2.5 ms and 5 ms (for low-gamma band oscillation). This division can achieve excellent recognition performance for clean speech. However, this short frame size can cause an accumulation of unnecessarily overlapping features and insertion errors as the noise level increases[2]. Previous studies indicated that the recognition performance is limited when the density of speech segmentation exceeds a certain level, which indicates that excessive speech segmentation is unnecessary[3]. Thus, we chose a quadrant division of theta and low-gamma oscillation as an optimal division size to capture various temporal changes of spectrum within speech signals.

Algorithm's recognition performance under different boundary condition

We used the boundary separation conditions ([-180°, -90°], [-90°, 0°], [0°, 90°], [90°, 180°]) because these conditions are prevalent in neuroscience studies that investigated the role of phase information in neuronal oscillations [4-7]. We have explored how the algorithm performs during phase re-parametrization of the boundaries. In this experiment, we considered equally spaced quadrant boundaries and shifted them clockwise by 20 degrees to create five different boundary conditions: ([-180°, -90°], [-90°, 0°], [0°, 90°], [90°, 180°]), ([-160°, -70°], [-70°, 20°], [20°, 110°], [110°, -160°]), ([-140°, -50°], [-50°, 40°], [40°, 130°], [130°, -140°]), ([-120°, -30°], [-30°, 60°], [60°, 150°], [150°, -120°]), ([-100°, -10°], [-10°, 80°], [80°, 170°], [170°, -100°]). We tested the algorithm performance under various noise levels. The result showed no significant differences between boundary conditions (Fig. S1). This result indicates that recognition performance is more related to the frequency of oscillatory reference and the thresholding parameter for detecting consonant regions.

Algorithm's recognition performance in the absence of a thresholding

We tested the algorithm performance in the absence of a threshold for various noise levels. We compared the recognition performance among the FFSR, nested oscillation (NVFS; theta-low gamma nested), and single frequency band oscillations (once with theta band oscillation and once with low-gamma band oscillation, which were employed as a primary oscillatory reference and a secondary oscillatory reference, respectively, in our study). We plotted the recognition accuracy (Fig. S2(a)) and the number of frames that were employed to segment consonant and vowel regions by each segmentation scheme (Fig. S2(b)). When speech is not strongly corrupted by noise, nested oscillation and gamma-band oscillation provides similar

recognition performance. This high performance can be explained by the relatively large number of frames that were employed by these two segmentation schemes to capture consonant and transition regions. As the noise increases, however, the performance of gamma-band oscillation significantly decreases compared with nested oscillation. This finding is attributed to the unnecessary number of frames that capture the vowel region, which eventually add redundant (noisy) information and cause insertion errors in the system, which reduces the recognition performance [2]. Considering the computational cost of speech recognition, gamma-band oscillation employs a larger number of frames than nested oscillation, which is computationally inefficient regarding their recognition performance. The theta-band oscillation indicated poor recognition performance because an insufficient number of frames is applied to segment consonant and transition regions, which creates difficulties in distinguishing different consonant types. As a result, a thresholding procedure is necessary to achieve high recognition accuracy and computational efficiency.

II. Supplementary figures

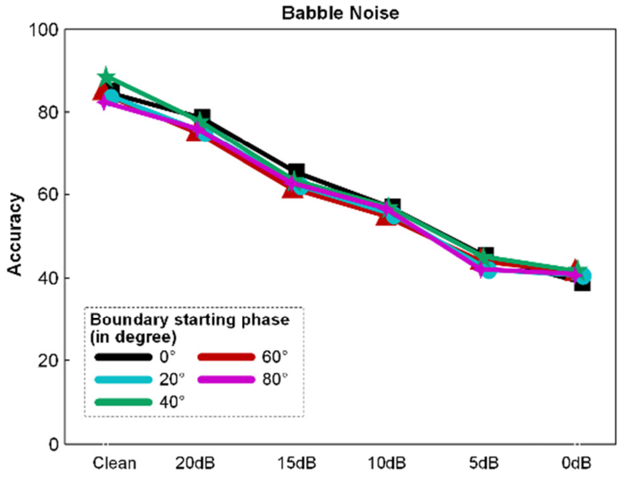

Supplementary Figure S1. Recognition performance between different boundary condition.

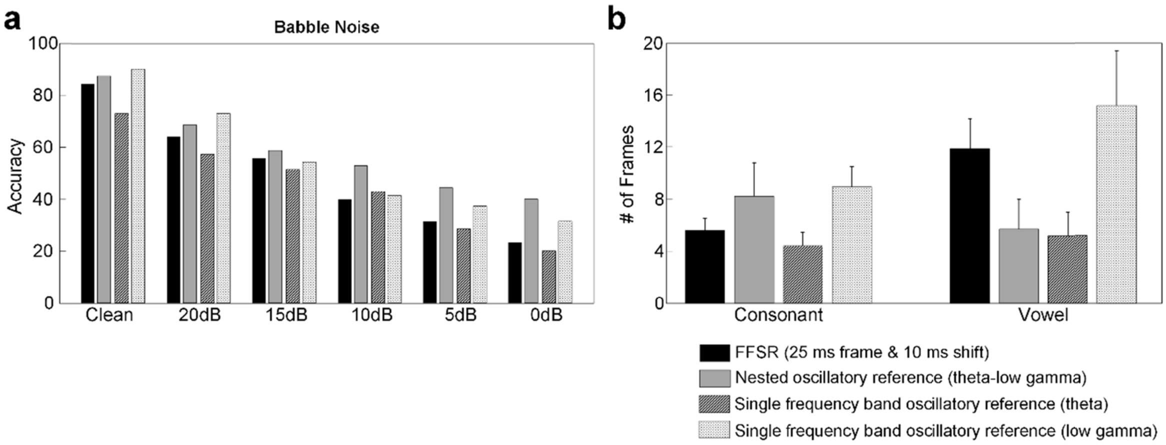

Supplementary Figure S2. Recognition performance among different thresholding schemes (a) Recognition results of four different segmentation schemes are evaluated. (b) Number of frames used to segment and consonant and vowel region in each segmentation scheme.

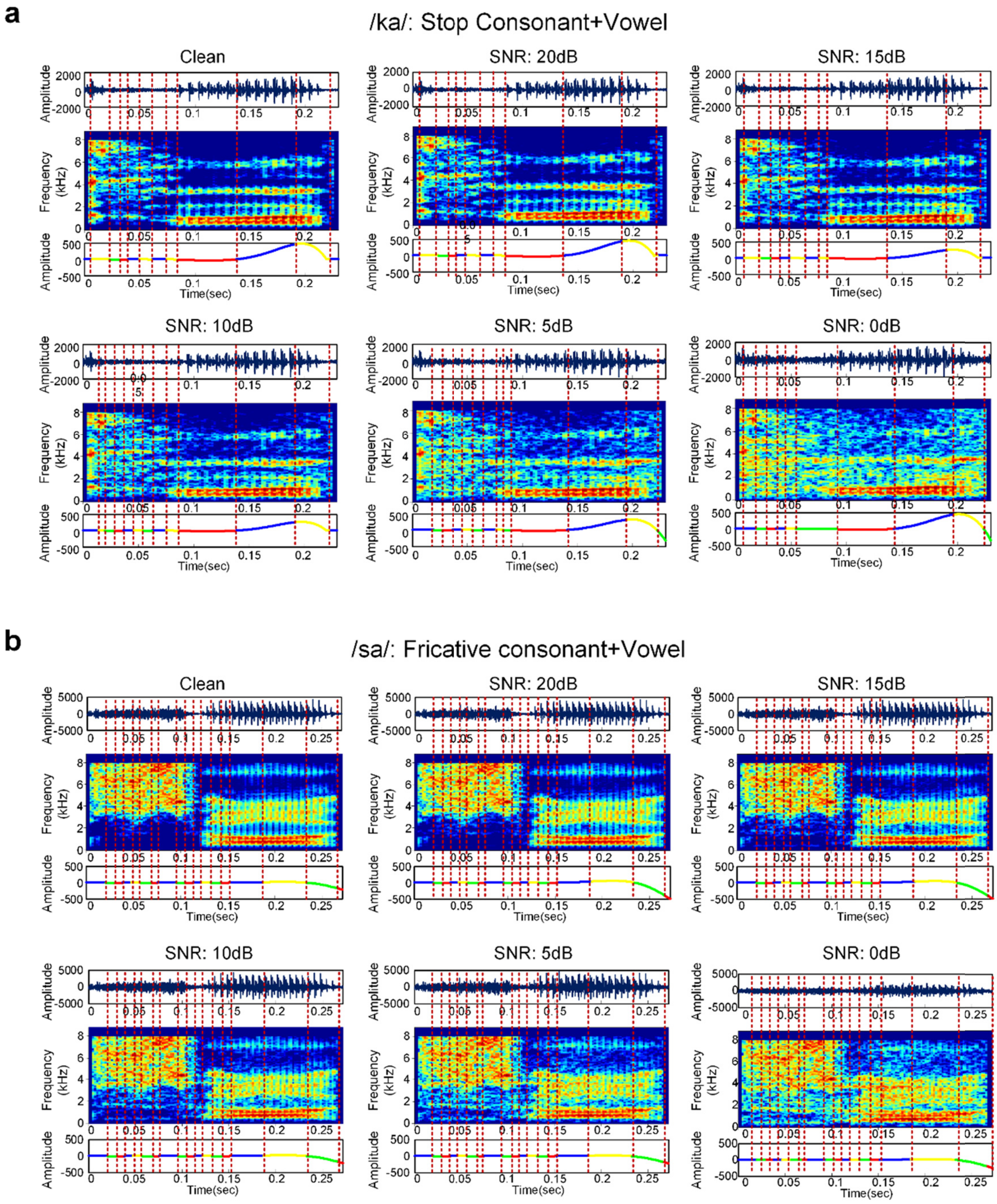
a
/ka/: Stop Consonant+Vowel
Clean
SNR: 20dB
SNR: 15dB
SNR: 10dB
SNR: 5dB
SNR: 0dB
Amplitude
Frequency (kHz)
Time(sec)
b
/sa/: Fricative consonant+Vowel
Clean
SNR: 20dB
SNR: 15dB
SNR: 10dB
SNR: 5dB
SNR: 0dB
Amplitude
Frequency (kHz)
Time(sec)

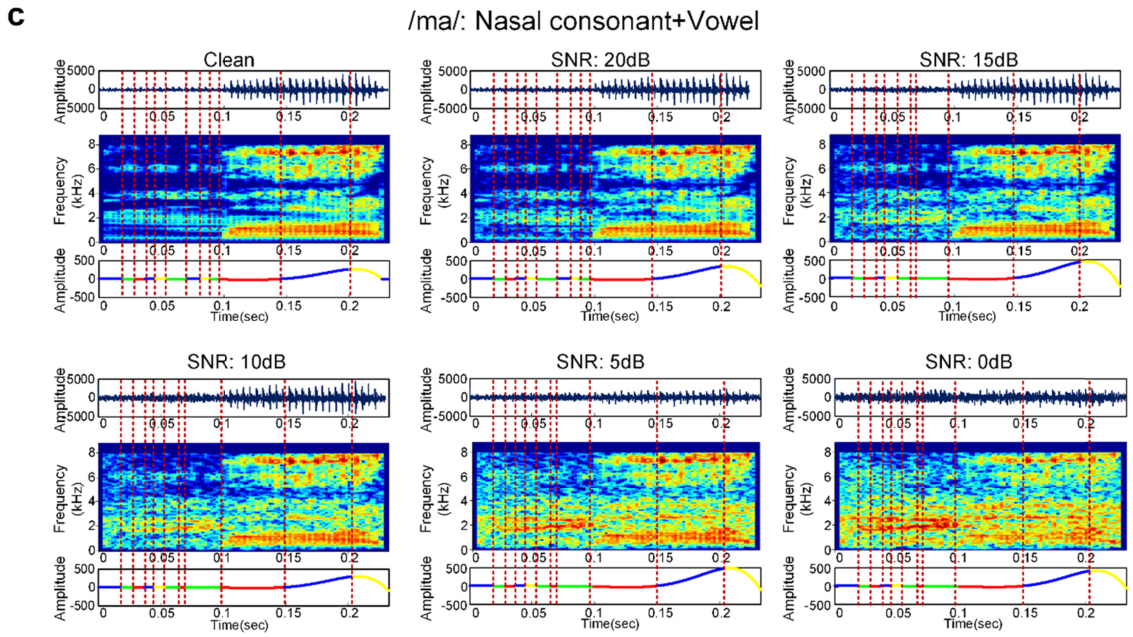

Supplementary Figure S3. Noise robustness of NVFS based speech segmentation on various SNR levels. NVFS scheme is applied to various syllable unit speech, which are composed of different consonant type: (a) stop consonant, (b) fricative consonant, and (c) nasal consonant. For all syllable samples, frame boundaries are decided by modulation rate of its envelope, which is short for fast modulation rate region (consonant and transition region) and relatively long for slow modulation rate region (vowel region). Frame boundaries are kept nearly constant over all SNR levels, showing robustness of NVFS based speech segmentation.

III. Supplementary References

1 Deller Jr, J. R., Proakis, J. G. & Hansen, J. H. *Discrete time processing of speech signals*. (Prentice Hall PTR, 1993).

2 Le Cerf, P. & Van Compernolle, D. A new variable frame analysis method for speech recognition. *Signal Processing Letters, IEEE* **1**, 185-187 (1994).

3 Loizou, P. C. *Speech enhancement: theory and practice*. (CRC press, 2013).

4 Kayser, C., Montemurro, M. A., Logothetis, N. K. & Panzeri, S. Spike-phase coding boosts and stabilizes information carried by spatial and temporal spike patterns. *Neuron* **61**, 597-608 (2009).

5 Panzeri, S., Brunel, N., Logothetis, N. K. & Kayser, C. Sensory neural codes using multiplexed temporal scales. *Trends in neurosciences* **33**, 111-120 (2010).

6 Cogan, G. B. & Poeppel, D. A mutual information analysis of neural coding of speech by low-frequency MEG phase information. *Journal of neurophysiology* **106**, 554-563 (2011).

7 Kayser, C., Ince, R. A. & Panzeri, S. Analysis of slow (theta) oscillations as a potential temporal reference frame for information coding in sensory cortices. *PLoS Comput Biol* **8**, e1002717 (2012).
